# Improved Micro-X-ray Fluorescence Confocal Imaging of Two-Dimensional Distribution of Arsenic Concentration in Cucumber Hypocotyls Using Synchrotron Radiation

**DOI:** 10.1021/acs.analchem.1c00579

**Published:** 2021-08-17

**Authors:** Imre Szalóki, Anita Gerényi, Ferenc Fodor, Gábor Radócz, Viktória Czech, Laszlo Vincze

**Affiliations:** # Institute of Nuclear Techniques, Budapest University of Technology and Economics, Budapest H-1111, Hungary; ‡ Department of Plant Physiology and Molecular Plant Biology, Eötvös Loránd University, Budapest H-1117, Hungary; † X-ray Microspectroscopy and Imaging Group (XMI), Department of Chemistry, Ghent University, Krijgslaan 281 S12, Ghent B-9000, Belgium

## Abstract

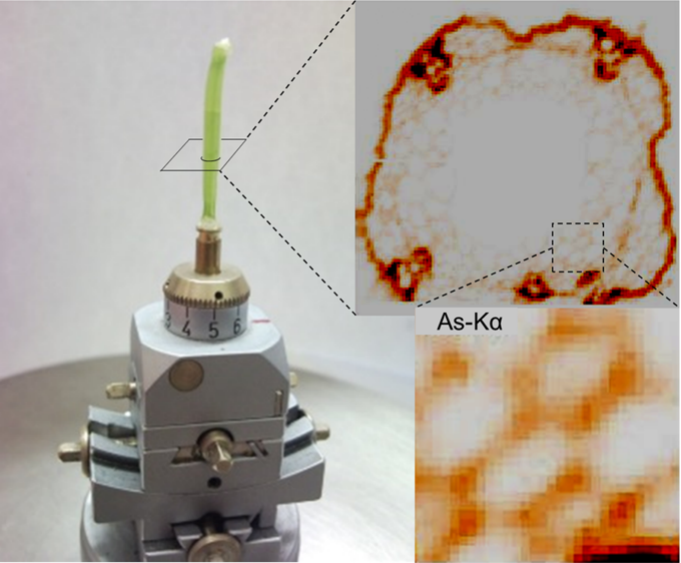

An optimized micro-X-ray fluorescence confocal imaging (μXRF-CI) analytical method has been developed to determine the 2D distribution of elemental composition in small (1–3 mm) biological objects at a 10–20 μm spatial resolution. Plants take up chemical elements from soil, and the vascular system transports them toward shoots. In order to obtain biochemical information related to this biological process, 2D distributions of chemical elements in roots and in hypocotyls of cucumber plants were analyzed by synchrotron radiation based on micro-X-ray fluorescence computer tomography and μXRF-CI techniques. The experiments were carried out at HASYLAB Beamline L of the DORIS-III storage ring in Hamburg, a facility that provided optimal physical conditions for developing and performing these unique analyses: high flux monochromatic synchrotron beam, X-ray optical elements, precision moving stages, and silicon drift detectors. New methodological improvements and experimental studies were carried out for applicability of lyophilized samples and cryo-cooling. Experimental parameters were optimized to maximize the excitation yield of arsenic Kα radiation and improvement of the spatial resolution of the μXRF-CI analytical method.

## Introduction

In several agricultural areas of the world, the arsenic concentration in groundwater is above the recommended health limit for drinking water that is 10 μg/L in the European Union.^1^ In some areas of Carpathian Basin, Hungary, the arsenic level reaches 180–200 μg/L. In this landscape, the origin of the abnormal As enrichment is primarily due to geochemical reasons, namely, the frequent occurrence of thermal water.^2^ This environmental condition leads to an arsenic concentration 2–10 times higher in the Carpathian Basin compared to other regions of the European continent.^3^ If groundwater is used for irrigation, then dissolved arsenic compounds can be redistributed into the soil from where the plant roots may take it up.^4^ The chemical forms of arsenic compounds are mainly arsenite (AsIII) and arsenate (AsV), and those could accumulate in plants.^5^ The arsenic compounds are translocated from the root to other tissues of the plants, such as leaves; therefore, these chemicals may influence metabolic processes. The accumulated compounds of arsenic and other toxic elements in the plants could enter the food chain, causing additional poisoning effects.^6^ Agricultural crops growing with arsenic contaminated water are a potential health hazard to the human populations and to the livestock if the contaminated plant material is used for animal forage purposes or human nutrition.^7^ For all of these reasons, the possible influence of toxicity on plant metabolism is one of the central problems of plant stress physiology, which is “How to obtain biochemical information on transport processes in plants?”

The stem of young dicot plants, the hypocotyl, is an appropriate target to study the element transport. Redox active nutrients and trace elements may suffer various chemical transformations during the uptake, assimilation, and sequestration.^8^ The cucumber is frequently used as a model plant; its hypocotyl was found to be especially sensitive to arsenate treatment in a certain stage of development.^9^ Arsenic may be taken up by plants through phosphate transporters especially when there is suboptimal phosphate supply. The pathway of element transport to the shoot is going within the xylem vessels in the hypocotyl. The flowing sap contains a mixture of transformed ions and organic compounds in a relatively stable chemical form. Therefore, the analysis of the hypocotyl and the xylem sap inside may provide a snapshot of the chemical transformation of the studied element, which may also shed light to physiological changes in the plant tissues.

In order to determine 2D/3D quantitative distribution of chemical elements in plant samples, various instrumental microanalytical methods are available such as LA-ICP-MS^10^ (laser ablation–inductively coupled plasma–mass spectrometry), LIBS^11^ (laser-induced breakdown spectroscopy), and EPMA^12^ (electron probe microanalysis). However, those analytical methods significantly destroy the biological structure or are suitable for surface analysis only. In contrast, micro-X-ray fluorescence (μXRF), micro-X-ray fluorescence confocal imaging computer tomography (μXRF-CT), and micro-X-ray fluorescence confocal imaging (μXRF-CI) techniques are more appropriate to determine the maps of 2D/3D distributions of chemical elements since these XRF methods may cause lower damage in the biological microstructure.

Synchrotron radiation is one of the most suitable exciting sources to achieve the best geometric resolution and detection limit, which is the excitation mode that is well suited to biological samples in the micrometer to millimeter size range,^13−15^ especially at the case of the full-field 3D XRF method.^16^ The synchrotron radiation (SR)-μXRF microanalytical technique can be combined with SEM–EDX (scanning electron microscopy coupled with energy dispersive X-ray microanalysis) and/or XANES (X-ray absorption near-edge structure) on the identically same plant sample item.^17^ This complex analysis provides an excellent synergy with simultaneous information on both quantitative distributions and chemical forms of the investigated elements. As an example for μXRF-CI and μXRF-CT based on SR, excitation was carried out for determination of chemical elements in *Daphnia magna* to disclose the influence of toxic metal ions on growth and reproduction of this biological item.^18^ Nanoprobe investigations with a high spatial resolution (∼180 nm) were done with a high X-ray photon flux (6 × 10^11^ photons/s) at the ESRF ID22NI beamline for the same type of sample.^19^

The distribution of elements’ concentrations and the chemical forms of ions in plants are of great importance for a better understanding of metabolic processes and the possible toxic effects of ions taken up from the soil. This chemical information can be obtained by XANES measurements, performing in vivo or in vitro mode with SR on cucumber hypocotyls.^20−23^ This type of investigation may offer relevant information on the quantity of As^V^ and As^III^ and reduction and oxidation processes.

The question is as follows: in what form and amount of various chemical compounds and ions enter the plant from the soil and how are they distributed in the tissues? This microanalytical research was motivated by researching an optimized μXRF mode for studying environmental problems arising from plant toxicity and bioaccumulation. The ultimate goal of this research project was to develop a highly sensitive micro-XRF analytical method using SR to determine the quantitative distribution of chemical elements, especially for arsenic in plant tissues for the purpose of physiological research of model organisms.

## Image of Biostructure: μXRF-CT and μXRF-CI

The μXRF-CI and μXRF-CT techniques using synchrotron radiation are two of the most suitable nondestructive microanalytical techniques to quantify 2D/3D distribution of chemical elements in biological samples. The μXRF-CT is based on sequential irradiation of the sample by a low-diameter (1–20 um) X-ray beam in an optional selected plane parallel to the X-ray beam. The characteristic X-ray photons emitted by sample atoms are detected using an energy dispersive X-ray detector. Scanning is performed along a selected sample plane, moving the sample with steps corresponding to the diameter of the beam. After each sequentially performed linear scan, the sample rotates 1–3° and the total linear scanning procedure is repeated until the sum of the angles of the sequentially performed rotation reaches 360°.

The concept of the μXRF-CI is based on the detection of characteristic X-rays emitted from a microsized sample volume determined by an overlap of two conical-shaped beams determined by the focusing optics of the X-ray source and the acceptance optics of the ED detector. The experimental setup of μXRF-CI can be realized primarily at synchrotron beamlines; however, it can be created with laboratory equipment as well using air-cooled X-ray tubes with an electric power of over 40–80 W and SD detectors.^24^ The geometrical resolution of the SR μXRF-CI setup can achieve an even submicron range depending on the energy of the exciting X-ray beam.^19^ The best limit of detection (LOD) values achieved were on the parts per million (relative) and femtogram (absolute) mass level depending on the atomic number of interest and the matrix composition. LODs were calculated using the expression  where *C* is the mass concentration of the analyzed element, variables *I*_B_, *I* represent the background and the fluorescence intensities respectively.^35^

**Figure 1:**
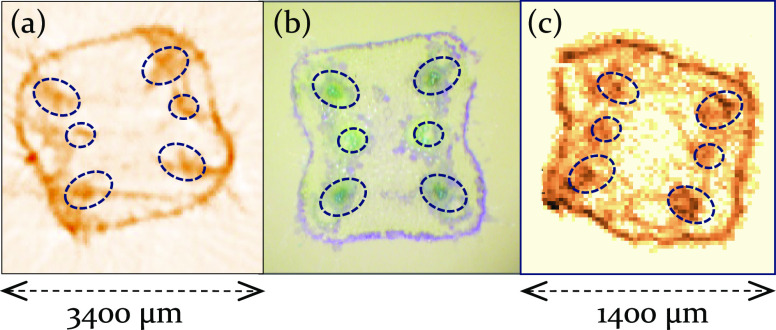
Comparison of reconstructed distributions of intensity of Zn Kα radiation emitted from cucumber hypocotyls measured by μXRF-CT (a) and μXRF-CI (c). Optical image of the cross section (b).

The differences between the reconstructed 2D images of μXRF-CT (a) and μXRF-CI (c) analytical techniques are demonstrated by two typical examples on Figure 1 where Zn Kα intensity distribution in a cross section of cucumber hypocotyls is plotted and an image recorded using an optical microscope (b) is shown. Both microanalytical experiments were carried out with the same diameter of the exciting X-ray beams, step size of the sample moving 20 μm, voxel sizes of 20 × 20 × 20 μm, and excitation energy of 20 keV.

The μXRF-CT picture contains uncorrected artifacts, while the μXRF-CI images have a better resolution despite identical technical conditions being used for both measurements. Comparison of basic analytical, technical, and measuring features of these two X-ray emission microanalytical methods are summarized in Table 1. Both analyses require mathematical reconstruction procedures for calculating the 2D distributions of the characteristic X-ray intensities and concentrations of involved chemical elements.

**Table 1:** Characterization of μXRF-CT and μXRF-CI

**μXRF-CT**	**μXRF-CI**
**X-ray Source and Optics**	
SR, X-ray tube; parallel beam; single bounced capillary (SBC); focal distance (20–50 mm); high-precision linear moving of SBC lens	SR, X-ray tube; focused beam; polycapillary (PC); focal distance (1–5 mm); high-precision linear moving of PC lens
**Mechanical Set Up and Spatial Resolution**	
linear motions (2D) + rotation of the sample, huge number of measurements	linear motions during 2D/3D measurement of the sample
**Setting Sample**	
pre-measurement to set the sample boundaries; complicated pre-setting procedure for the right sample position	simple procedure to determine the volume of μXRF-CI; easy pre-setting of the sample position
**Measuring Conditions**	
cryo-cooling by LN_2_ (liquid nitrogen) stream jet, possible in situ study. Sample preparation: dry-freezing, hydration method.	sample cannot be cooled by LN_2_ stream jet. Sample preparation: dry-freezing, hydration method.
**Post-measurability for Lost Spectra**	
possible	possible
**Reconstruction**	
Compensating artifacts by mathematical procedures: filtered back projection	no artifacts, depth-dependent absorption correction is necessary
**Quantitative Evaluation**	
FPM^32^ (fundamental parameter method) approximations are necessary for quantitative determination of chemical elements	possible, absorption correction of the matrix for excitation beam and for characteristic radiations^32^

## Experimental Section

Cucumber (*Cucumis sativus* L. cv. Joker 1) was used as a model plant grown in modified Hoagland’s nutrient solution: 1.25 mM KNO_3_, 1.25 mM Ca(NO_3_)2, 0.5 mM MgSO_4_, 0,25 mM KH_2_PO_4_, 11.6 μM H_3_BO_3_, 4.6 μM MnCl_2_ 4H_2_O, 0.19 μM ZnSO_4_·7H_2_O, 0.12 μM Na_2_MoO_4_·2H_2_O, and 0.08 μM CuSO_4_·5H_2_O. Moreover, Fe was added into the solution in the form of FeCl_3_ (0.01 mM). The light period for the model plants in the growth chamber was set to 14/10 h light/dark with a temperature of 26/22 °C day/night, and the humidity was kept at 70–80%. The germination period was 30–32 h and after that, the plants were grown for 5 days in the standard nutrient solution. After the pre-growth period, the seedlings were transferred to a low phosphate nutrient solution (0.002 mM KH_2_PO_4_). Twenty-four to twenty-eight hours before the in vivo measurements, the plant samples were transferred again to normal Hoagland’s nutrient solution supplemented with 100 μM arsenic in the form of As(V).

**Figure 2:**
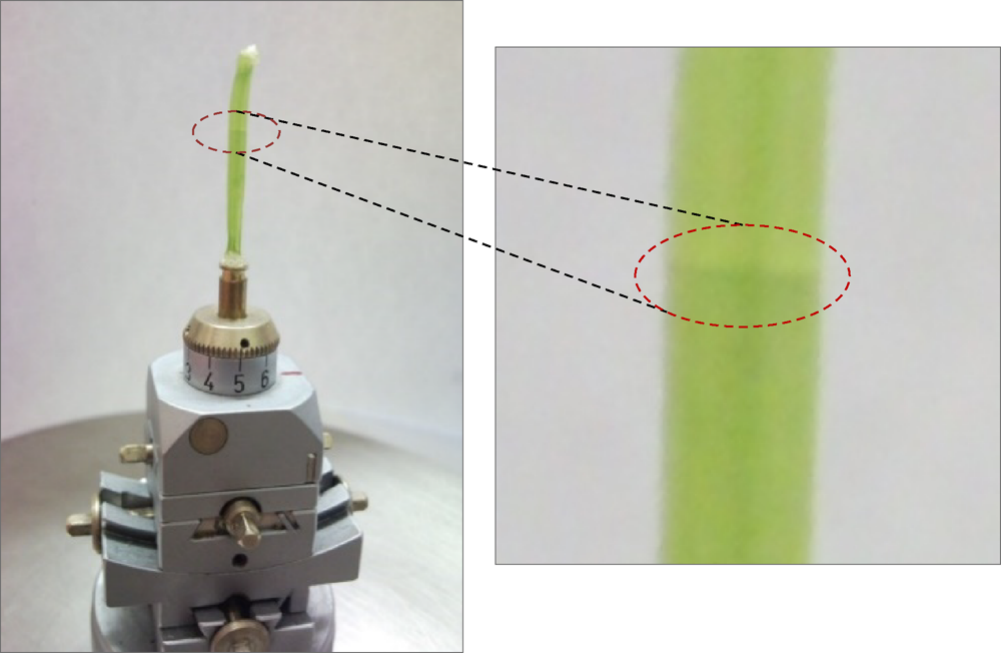
Cucumber hypocotyl sample damaged by the synchrotron beam (17 keV) used for the excitation. The irradiation duration was 1 h at room temperature (22 °C).

Both μXRF-CT and μXRF-CI experiments were carried out at the HASYLAB Beamline L of the DORIS-III storage ring Hamburg, Germany. The construction and available equipment of this hard X-ray beamline fit well to perform μXRF experiments with white or monochromatic excitation using mono- or polycapillary optical elements. In μXRF experiments, a Ni/C multilayer monochromator was applied to obtain a quasi-monochromatic beam in an energy range of 12.5–22 keV. The relative spectral bandwidth was Δ*E*/*E* ≈ 1.8%. For both types of μXRF experiments, the indispensable requirement is the accurate setting of sample position. Moreover, a stable geometrical position is necessary in each measuring phase, and for this reason, the plant samples that were mounted using glue on top of a glass capillary or brass holder were fixed into a 5-axis goniometer head (Figure 2). The goniometer was fitted to a 3D moving stage that provided the sample positioning by motorized linear motion with 0.1 μm precision and rotation in 0–360° during μXRF-CT.

The μXRF-CI set-up contained two polycapillary half-lenses (PC) for focusing the exciting SR and the secondary X-ray radiation. These optical elements were designed and manufactured by X-ray Optical Systems Inc. An angle of 90° was set for alignments of μXRF-CT and μXRF-CI between the axes of primary and secondary X-ray beams. For measuring the secondary X-rays, VORTEX SD detectors were applied (SII Nano Technology USA Inc.). The active area of SD detector crystals was 50 mm^2^, and the thickness of the sensitive layers was 350 μm. The complete measuring processes and the spectra evaluation procedures were controlled by the software package MicroXRF2.^25−27^

In order to decrease the scattered radiation in μXRF-CI experiments, lyophilized samples were used having water content near zero. Moreover, the confocal setup significantly limits the detection of scattered radiation emitted from voxels that are located in the detector channel since the solid angle of the acceptance polycapillary optics is narrow. In contrast to μXRF-CI, the μXRF-CT allows the detection of higher flux of the scattered radiation due to the large solid angle of the energy dispersive X-ray detector. Due to this disadvantageous character of the μXRF-CT setup, the excitation energy of the synchrotron radiation was set to 13.5 keV.

### Beam-Damage Effect

Biological samples consisting of large water-containing soft tissues are sensitive to intense synchrotron radiation. Due to the absorption of SR in the sample material, the local temperature increases and the water in the tissue may boil, causing damage and distortion of the original cell structure. This effect prevents a realistic determination of the quantitative 2D distribution of chemical elements in the sample tissues. A typical example of the radiation damage effect is shown in Figure 2, where a cucumber hypocotyl sample is shown, irradiated with SR (*E*_0_ = 17 keV) for 1 h at room temperature. In this analytical field, several different possible methods are known to avoid or reduce the radiological effect in biological samples. (i) The distortion of the biological microstructure of the in vivo sample can be reduced by cryogenic cooling. This procedure requires a continuous jet of LN_2_ stream^28,29^ blowing to the sample and to its close environment. (ii) The freeze-drying method provides losses of the complete water content of the sample while its temperature is kept below 0 °C. In our experience, the lyophilized hypocotyl of cucumber plants had a very stable structure during the μXRF measurement due to the zero content of the water. (iii) The water content of the sample is chemically removed by the HMDS (1,1,1,3,3,3-hexamethyldisilazane) technique, and the missing water in the sample tissues is replaced by resin.^30^

The easiest sample preparation method for in vivo measurement is when a piece of hypocotyl or root is cut from the original living cucumber plant and both end of this sample is closed by wax or watertight glue against the loss of liquid from the sample, which kept the in vivo status of the sample.

### μXRF-CT Experiments

The μXRF-CT setup requires an SBC lens^31^ since this optic has a significantly larger focal distance (30–50 mm) than PC lenses (1–5 mm) and produces a quasi-parallel SR beam. The long focal distance allows the use of cryo-cooling by an LN_2_ stream jet. The SBC lenses focus the SR beam with a smaller convergence than PC lenses.

In μXRF-CT experiments, the samples were measured in their original biological state using LN_2_ cooling in order to limit the beam-damage effect. The cryo-stream nozzle was fixed at about 45° to the plane determined using exciting and excited X-ray beams. The sample temperature was approximately 100 K.^28^ The sample has a strong self-absorption to the characteristic radiation of light chemical elements that are on the opposite side of the sample from the detector. To compensate for this phenomenon, obtain a higher spectral signal, and reduce the measurement time, two SD detectors were applied on opposite sides of the sample. The preference of this setup is that the quantitative distributions of chemical elements can be reconstructed on the basis of the sum of two XRF spectra and the XRF intensities of light elements (13 ≤ *Z* ≤ 20) can be detected by an improved statistic compared with what is possible with only one detector. Due to the moisture content of the air and the low temperature of the sample, on the sample surface, icing is going on, which results in artifacts on the reconstructed tomographic images.

### μXRF-CI Experiments

Higher detected intensities can be achieved with PC lenses than with SBC lenses. However, use of polycapillaries does not allow cryogenic cooling due to the small working distance of the PCs (1–5 mm) since the LN_2_ vapor flow also cools the material of PC lens and therefore is of high risk of breakage.

**Figure 3:**
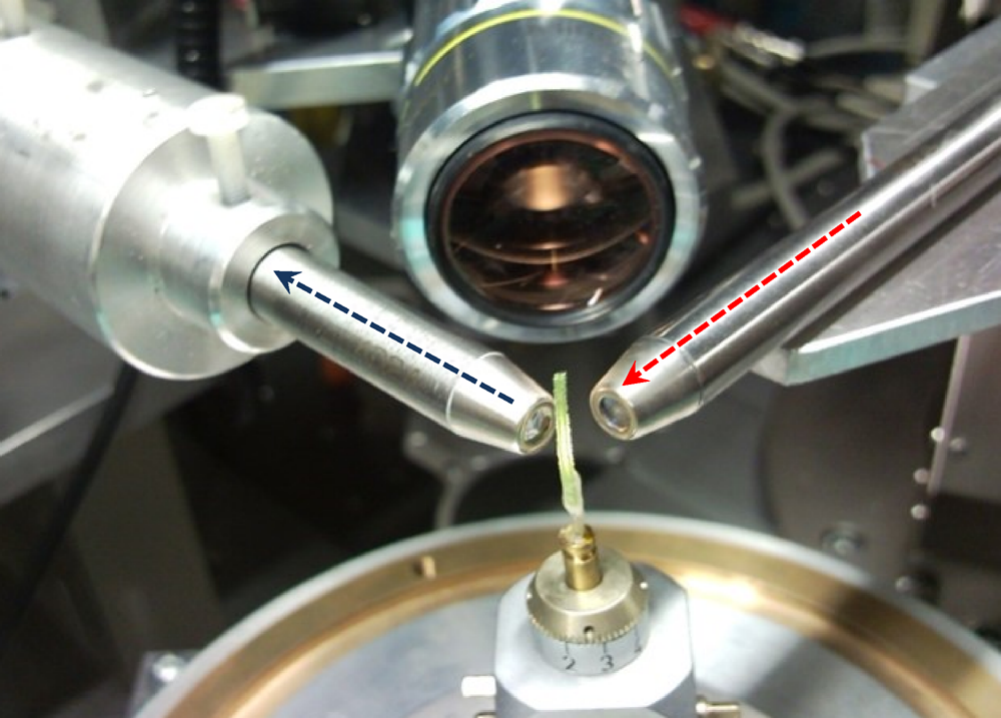
Setup of μXRF-CI on a freeze-dried cucumber hypocotyl at the HASYLAB Beamline L.

A focused synchrotron beam can damage biological samples in a long-term duration (5–10 h) of cyclic scanning experiments, so samples were freeze-dried before the experiments. The energy of the incident SR was selected in a range of 12.5 22 keV. The focal distance of the PC was 8 mm with a 10 μm diameter of the focal spot. The acceptance capillary mounted in front of the SD detector had a shorter focal distance (∼2.5 mm), but the diameter of the focal spot was also about 10 μm (Figure 3). The geometric resolution of the entire setup was found to be 13 × 17 μm at an energy of 14.0 keV.

The measuring time of a single voxel was set to 5–10 s depending on count rates of the measured intensities, which were normalized to a constant DORIS current of 100 mA. The distance between two neighboring pixels, i.e., step size of the confocal scanning, corresponded to the FWHM of the Gaussian distribution of the synchrotron beam flux. The simplest measuring strategy is when movement of the sample and measurement are separated in time, i.e., the “stop-measurement-storage-start-move-stop”.

**Figure 4:**
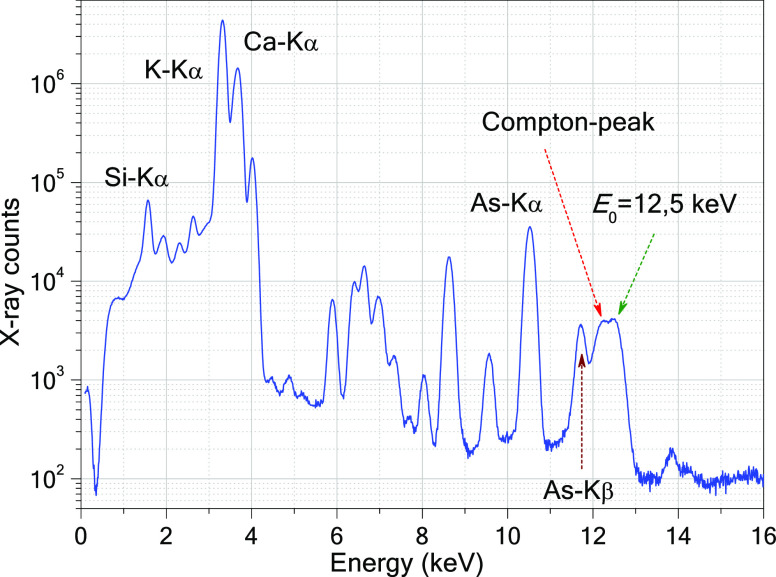
Sum spectrum of As in cucumber hypocotyls measured in the μXRF-CI setup.

The duration of the sample stopping and starting procedures can be saved with the so-called “dynamic scanning mode”.^18^ In this algorithm, the acquisition of XRF spectra and data storage are performed cyclically while the sample is moving continuously without stopping. XRF spectra were evaluated by the AXIL code embedded into the MicroXRF2 software package. The initial values of spectra evaluation parameters were determined by fitting the sum spectrum obtained as the summation of the measured spectra (Figure 4).

### Optimization of Measuring Conditions

Since the samples contained arsenic in a trace concentration range of 10^–5^ – 10^–4^ g/g, therefore, measuring conditions had to be designed to maximize the detected As-Kα intensity. In order to achieve the optimized condition, the SR energy has to be as close as possible to the binding energy of K electrons. The calculation of the intensity of arsenic fluorescence radiation (As-Kα) can be approximated by the FPM model of XRF analysis.^32^ FPM models consider the fundamental atomic parameters and energy distribution of flux density of the exciting SR beam *I*(*E*), which in these experiments was quasi-monochromatic at energy *E*, produced by a Ni/C double multilayer monochromator system that consisted of 100 layers.^33^ The FWHM_p_(*E*) functions of primary (p) polycapillary half-lenses were determined experimentally by Falkenberg et al.^34^ They found that FWHM_p_(*E*)/*E* varied between 1.65 and 2.19% in an energy interval of 8–21 keV and the absolute values of FWHM_p_(*E*) were in a range of 150–460 eV.

The energy-dependent intensity distribution of the characteristic X-ray radiation related to atomic number *Z* can be characterized by the Gaussian function in eq 1 where the energy resolution FWHM*_p_*(*E*) of the exciting beam is considered. In order to simplify the calculations of *I_Z_* based on relationship 1, the matrix effect and the second-order excitation process were neglected. The sample was supposed to be a thin layer of a pure element.
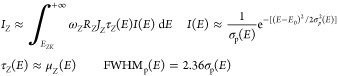
1

In eq 1, variable *Z* is the atomic number, ω*_Z_* is the fluorescence yield, *R_Z_* is the radiative rate of the relaxation process, and *J_Z_* is the absorption jump ratio. The photoelectric cross section τ*_Z_*(*E*) can be substituted by the mass-photo-absorption function μ*_Z_*(*E*).^35^ This function can be approximated by empirical formula 2, where *E_ZK_* is the binding energy of the *K* atomic shell belonging to atomic number *Z*. Parameters *a_Z_* and *b_Z_* are empirically determined constants.^35,36^
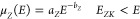
2

Using function 2, the excitation function can be derived as eq 3
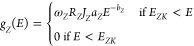
3

Eq 4 gives the characteristic intensity, complementing it with the energy-dependent transmission function *T*_p_(*E*) of the primary polycapillary half-lens.^37^ Parameter *c*_1_ is a multiplication factor that is proportional to the photon flux of the exciting SR radiation and to the excited area of the thin sample.
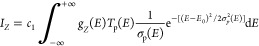
4

The energy-dependent transmission of the polycapillary lens can be mathematically described by the multiplication of exponential- and power-type functions.^37,32^

**Figure 5:**
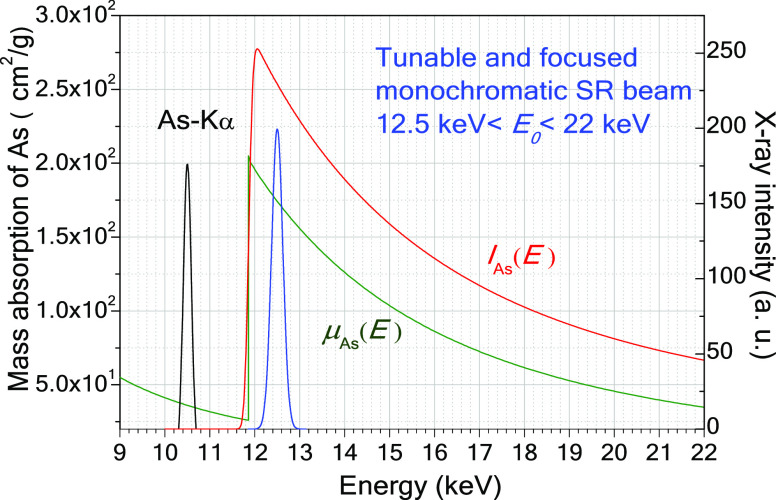
Mass absorption coefficient of arsenic versus energy (green). Energy distribution of the synchrotron beam (blue). As-Kα characteristic intensity *I_As_*(*E*) (red). *E*_0_ is the centrum of the Gauss-type function.

On Figure 5, the numerical values of the As-Kα characteristic intensity *I*_As_(*E*) calculated by eq 4 are plotted (red). The maximum of this curve (∼12.1 keV) is just found on the high-energy side of the K-edge of the arsenic mass absorption function (green).

The energy of the SR beam is worth setting to about 12.5 keV since As-Kα intensity is at this excitation energy that is five times higher than at an energy of 21 keV. Selecting a lower energy than 12.5 KeV, the spectral contribution of the Compton scattering overlaps with the As-Kα and As-Kβ peaks (see Figure 4), especially in the case of the matrix consisting of low-*Z* elements.

Further possible improvement of detected information that originated from μXRF-CI measurement is possible to increase the frequency of sampling sites. The sizes of the confocal volume (probing volume) are used to define the FWHM of the spot created by the overlap of the primary SR beam and the detector channel.^38^

**Figure 6:**
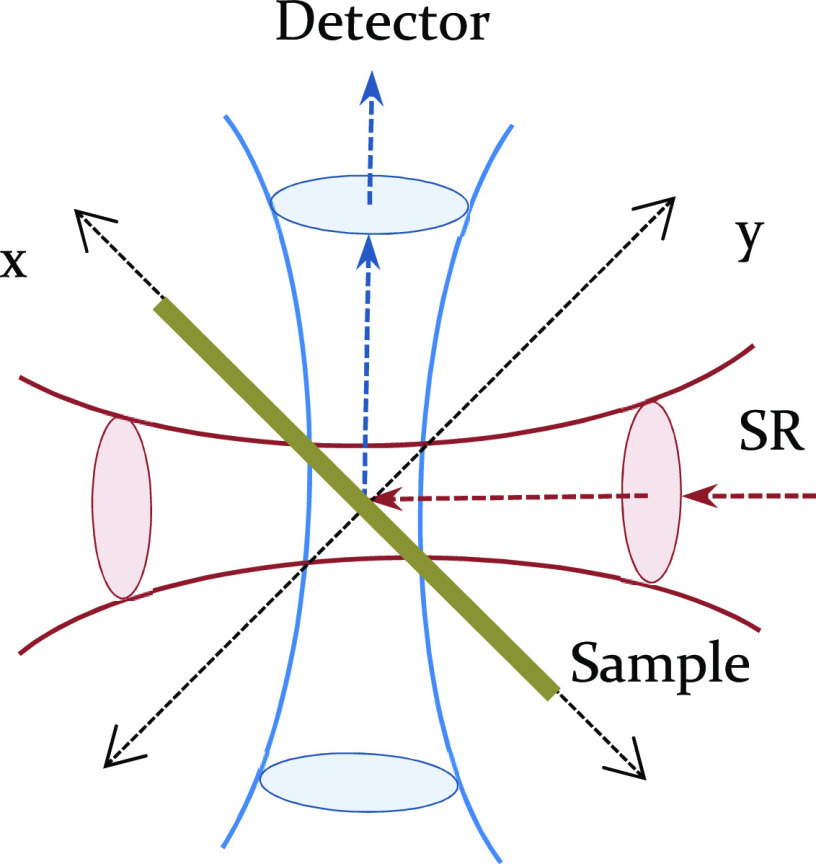
Theoretical setup for optimization calculations of measuring conditions, where the thin sample consists of a single pure element.

Let us suppose that the size of the confocal area in the *x*-direction (Figure 6) at the characteristic X-ray energy of chemical element *Z* is *A_Zx_*. The step size of the linear scanning was set to be equal to this value. This parameter determines the geometrical resolution of μXRF-CI along the *x*-axis. In order to describe this relationship, a new factor given in eq 5 was added to eq 4, calculating the lateral intensity distribution of the detected characteristic radiation.
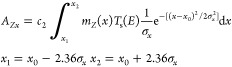
5

In the first term in eq 5, *c*_2_ is the proportional factor and function *m_Z_*(*x*) is the distribution of mass of the pure element sample with atomic number *Z*. Finally, both the geometrical and energy-dependent intensity distribution can be considered in eq 6, which was derived from eqs 4 and 5.
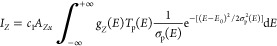
6

**Figure 7:**
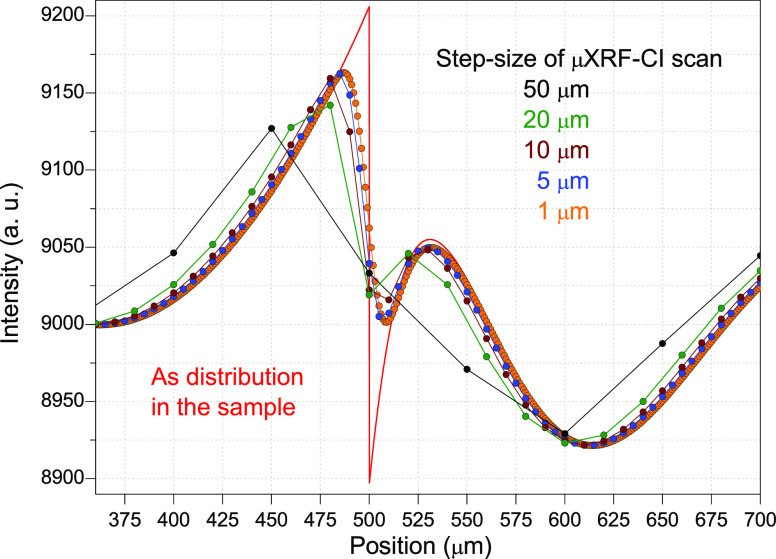
Result of the scan strategy of the μXRF-CI step size in a range of 1–50 μm. The FWHM of the monochromatized SR beam was 15 μm.

A new question arises, how does the lateral resolution in the map of the characteristic X-ray intensity depend on the variation of the step size? Arbitrarily, selected nonhomogeneous quantitative distribution of arsenic was assumed (Figure 7, red curve) and the characteristic intensity was calculated by eq 6.

The result of the calculated line-scan is plotted at different step sizes as 1, 5, 10, 20, and 50 μm, demonstrating that the similarity between the calculated and original distribution of arsenic quantity increasingly becomes better with refinement of the step size.

## Results

**Figure 8:**
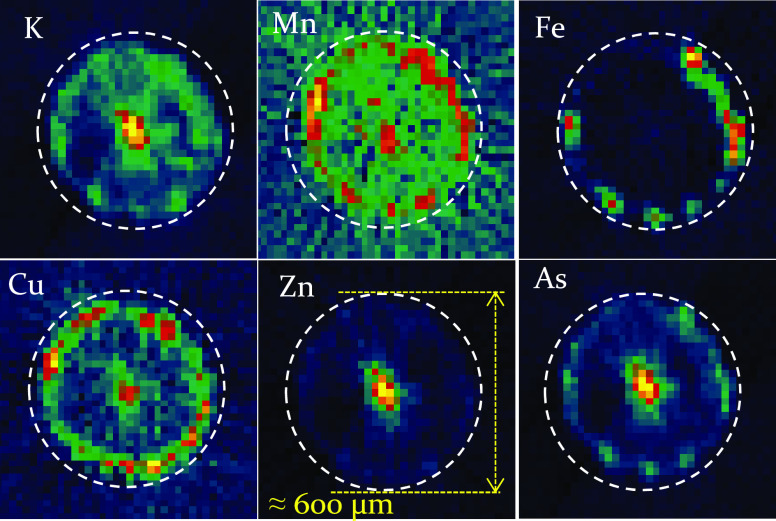
Reconstructed Kα intensity distributions of chemical elements in cucumber roots determined by the μXRF-CT technique.

The μXRF-CT analyses were performed in vivo on small-diameter (∼600 μm) sections of cucumber roots by cryo-cooling to reduce radiation damage. In this part of the plants, xylem- and phloem-type channels have not yet been separated (Figure 8). The elements K, Mn, Cu, Zn, and As are concentrated mostly in the xylem/phloem channel located in the central cylinder of the root.

The only exception is Fe, but this element is accumulated in the epidermal cells similarly to Cu and Mn. The characteristic intensities of Mn and Cu were too low, which caused a noisy image in spite of the filtered back-projection reconstruction.

In order to draw relevant biological conclusions, a higher spatial resolution of the quantitative distribution of chemical elements is required. Achieving this goal, optimization of measuring conditions was performed to improve the geometrical resolution of element maps: (i) tuning the excitation energy of the SR beam, (ii) setting the sampling frequency, (iii) setting the acquisition time, and (iv) varying the size of voxels. After the optimization steps, the procedure of the μXRF-CI technique has become capable of visualizing a single biological cell.

**Figure 9:**
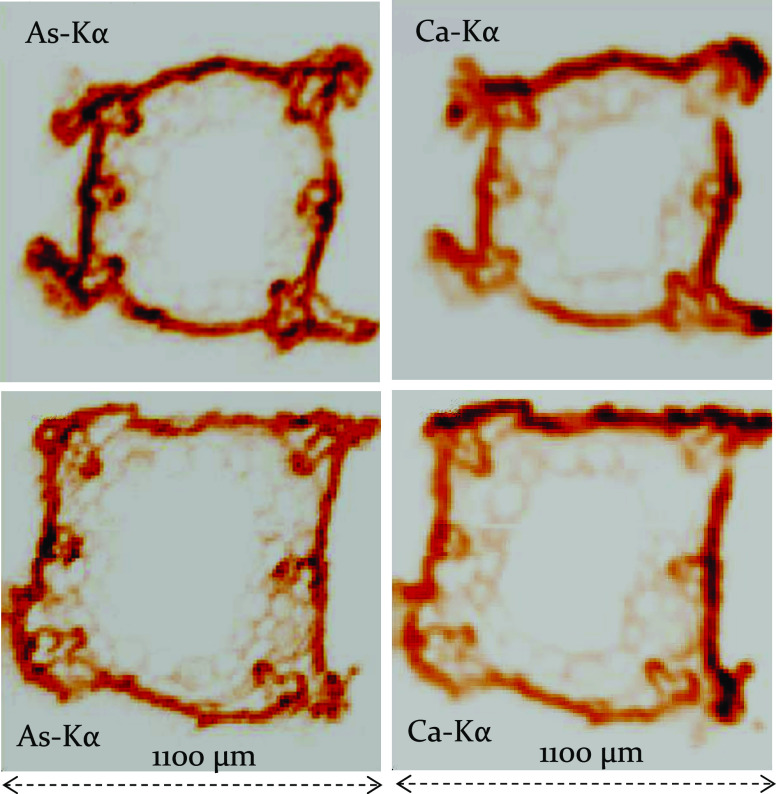
Ca-Kα and As-Kα intensity maps in cross sections of two hypocotyl samples. The nutrient solution contained 100 μM As(V).

**Figure 10:**
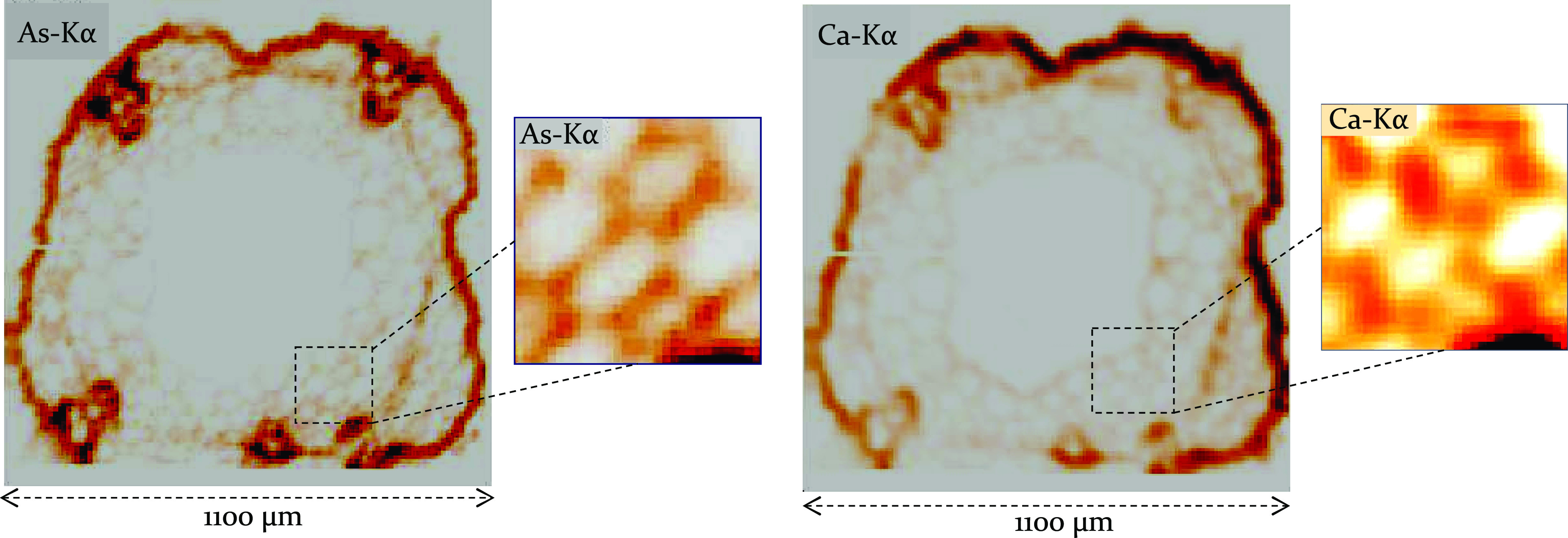
Distributions of As-Kα and Ca-Kα intensities within a cross section of a lyophilized hypocotyl sample. The nutrient solution contained 100 μM As(V). The excitation energy of the synchrotron beam was 12.4 keV, the step size of the confocal imaging was set as 5–10 μm, and the beam size was 20 μm.

The results of μXRF-CI experiments carried out under optimized measuring conditions described in the previous section are plotted on Figures 9 and 10, illustrating the intensity distribution of Kα characteristic lines of Ca and As in an optionally selected cross section perpendicular to the vertical axis of the hypocotyl sample. Figures 9 and 10 show that most of the arsenic contents are concentrated in the channels and along the edge part of the sample.

The improper lyophilization may result in the significant distortion of the biological structure in both elements precipitating on the cell walls and being concentrated in the dried sample. Other chemical elements added to the nutrient solution (K, Mn, Zn, and Cu) had a similar behavior. In order to achieve the highest lateral resolution in the 2D fluorescence maps under current geometrical conditions, the sweep scans were performed with a 10 μm step size (voxel size) with the SR beam having a diameter of 20 μm. The spectra acquisition time per voxel was increased up to 10 s, and those conditions allowed reaching few hundred cps/point for the arsenic Kα signal.

To maximize the counts of the arsenic lines, the excitation energy was set with multilayer monochromators to 12.5 keV, very close to the arsenic K-shell absorption energy. Instead of 3D scanning, only 2D horizontal maps were measured because of the high degree of similarity of tissue structures of hypocotyl within the longitudinal direction of the hypocotyl. Improving the geometric resolution of the elemental maps, a step size/voxel of 10 μm was used, while the diameter of the exciting SR beam used for the μXRF-CI experiment was kept at 20 μm. Corresponding to the calculated results on Figure 7, the reduced step size may improve the lateral resolution of the scan. The measuring time per point was set to several seconds, allowing a detection level of a few hundred counts for arsenic.

The result of the confocal imaging shows that As was transported into the individual cells of the hypocotyl. Not only the xylem vessels but also the epidermal cells and the parenchyma contained As. The large-diameter cells of the xylem vessels are located in the corners of the hypocotyl on Figures 9 and 10. It can be concluded that this new experimental setup was successful in visualizing the adsorption of As in cells: (i) high excitation probability of the As-Kα line with the selection of the excitation energy and (ii) lower step size than the beam size.

The tomographic reconstruction and confocal micro-XRF analyses of cucumber roots and hypocotyl samples revealed the internal biological microstructure of the investigated samples, and the obtained 2D and 3D elemental distributions demonstrated the possibility of localizing the toxic metal-enriched microscopic regions.

### Quantification

A new quantitative algebraic reconstruction model for confocal micro-X-ray fluorescence data has been developed for biological samples for the μXRF-CI technique using an SR excitation beam.^32^ The theoretical model is based on a generalized FPM mathematical description of calculated concentrations of chemical elements in each individual voxels of the sample. The numerical solution of the system of equations is based on an iterative numerical approach to calculate the 2D distribution of the concentration of the detected chemical elements along an optionally selected plane in the sample.

**Figure 11:**
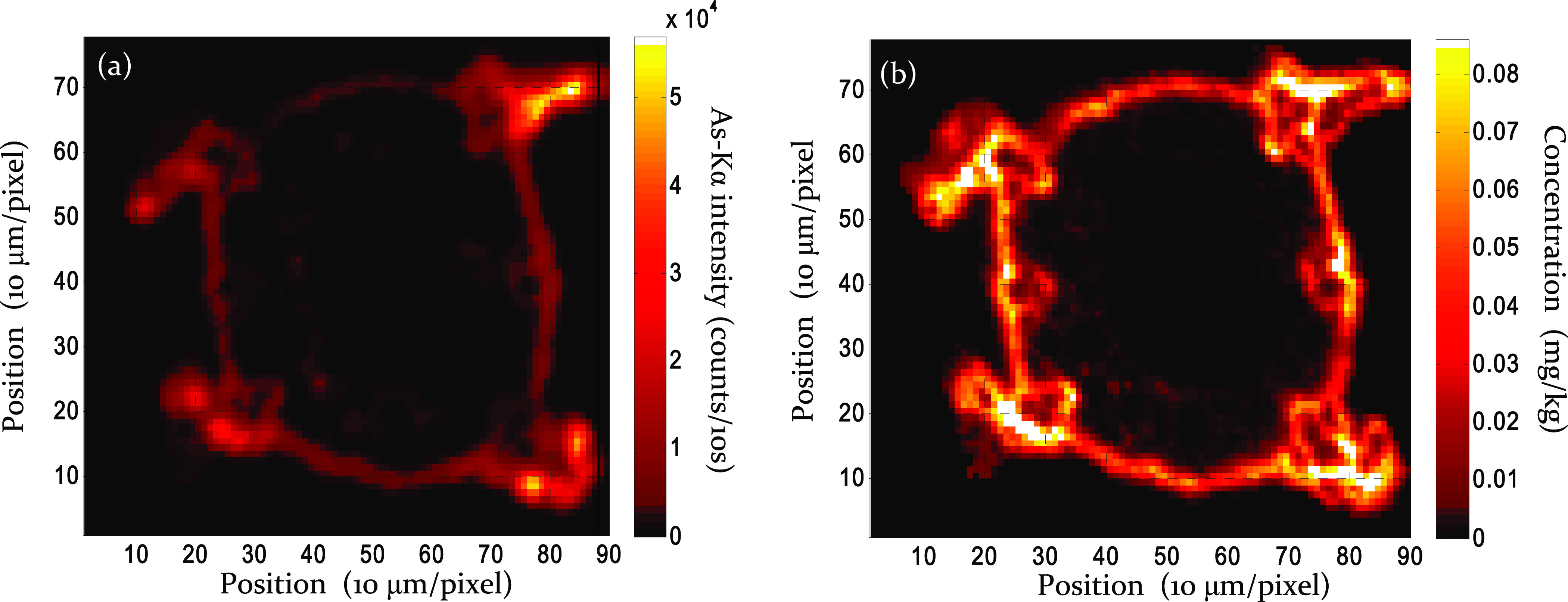
Distribution of K-Kα intensity (a) and FPM calculated concentration (mg/g) in the hypocotyl cross section (b). The nutrient solution contained 100 μM As(V).

The model considers the absorption effects for both the exciter and the excited X-ray radiations in every voxel along the paths of both X-ray beams. The validated model can be solved numerically by a new type of iterative algorithm developed in an MATLAB programing environment for evaluation of 2D confocal imaging using monoenergetic synchrotron radiation for the excitation of the sample elements. This FPM model was applied for calculation of element’s concentrations in cucumber hypocotyl samples. An example for the quantification calculation performed by the FPM model is demonstrated in Figure 11. Subfigure (a) is the map of intensity distribution of K-Kα in a hypocotyl sample, and (b) exhibits the FPM-calculated concentrations of K in mg/g unit.

## Conclusions

Plants are capable of uptaking chemical elements, accumulating them in tissues, and transporting them in the xylem sap from roots to the leaves. The toxic elements, especially arsenic and its compounds, may strongly influence the metabolic processes of plants, and those chemicals may constitute a risk factor for human health by entering the food chain. Cucumber is used as an appropriate model plant as an indicator of the bioavailability of one of the toxic elements, arsenic, in the soil. To obtain information about the ion-transport mechanisms in cucumber plants, optimized μXRF-CT and μXRF-CI microanalytical techniques were developed to determine a quantitative map of the chemical elements in the hypocotyl part of cucumber plants. The experiments were carried out at the Beamline-L at DESY-III storage ring in Hamburg, a facility that offered optimal physical conditions for development of these 2D analyses: a high flux of synchrotron beam, X-ray optical elements, precise moving stages, and cryo-cooling device. Optimization of some experimental parameters (exciter energy and step size of mapping) is based on a simple theoretical and numerical calculation for maximization of the excitation efficiency for arsenic Kα characteristic radiation by tuning the energy of the exciter SR beam. The results of this new measuring procedure can be utilized for any other chemical elements depending on the energy range in which the SR beam energy can be tuned at the synchrotron beamline. Another analytical benefit of this project is the improvement of the spatial resolution of the mapping procedure, applying the refinement of the step size of the mapping procedure. The top result of the research was that it became possible to visualize the arsenic distribution in individual plant cells (50–90 μm) of hypocotyls of the studied cucumber plants despite the fact that the diameter of the exciter X-ray beam was only between 10 and 20 μm. The method we have developed is likely to be adaptable to SR beamlines where the beam size is significantly smaller, possibly on the sub-micrometer order.
